# Ischemic Bowel Syndrome in Patients with Spinal Cord Injury: A Nationwide Study

**DOI:** 10.1371/journal.pone.0169070

**Published:** 2017-01-05

**Authors:** Chih-Wei Tseng, Cheng-Li Lin, Yu-Tso Chen, Long-Bin Jeng

**Affiliations:** 1 Division of Allergy, Immunology and Rheumatology, Taichung Veterans General Hospital, Taichung, Taiwan; 2 Management Office for Health Data, China Medical University Hospital, Taichung, Taiwan; 3 College of Medicine, China Medical University, Taichung, Taiwan; 4 Division of Gastroenterology and Hepatology, Department of Internal Medicine, Feng Yuan Hospital, Ministry of Health and Welfare, Taichung, Taiwan; 5 Graduate Institute of Clinical Medical Science and School of Medicine, College of Medicine, China Medical University, Taichung, Taiwan; 6 Department of Surgery, Organ Transplantation Center, China Medical University Hospital, Taichung, Taiwan; University of Toronto, CANADA

## Abstract

**Purpose:**

The aim of this study was to determine whether spinal cord injuries (SCI) is associated with increased risk of ischemic bowel syndrome (IBS) in an Asian population by analyzing data from the National Health Insurance Research Database (NHIRD) in Taiwan.

**Methods:**

Patients aged ≥20 years in the inpatient database with newly identified SCI from 2000 to 2011 were selected as the SCI cohort. For the non-SCI cohort, patients were selected based on a 1:4 risk-set sampling. Hospitalization with a new diagnosis of IBS during the follow-up was the main outcome measure. We used the standard univariable and multivariable Cox proportional hazard regression models to determine adjusted subhazard ratios (SHR) and 95% confidence interval (CI) in the SCI and non-SCI cohorts.

**Results:**

Patients with SCI were at significant risk for IBS, with an adjusted SHR (aSHR) of 1.25, 95% CI = 1.04–1.51. Multivariable analysis showed individuals with SCI were associated with a greater risk of IBS than individuals without SCI among males (aSHR = 1.47, 95% CI = 1.16–1.86), all age groups (≤49 y: aSHR = 2.15, 95% CI = 1.24–3.74; 50–65 y: aSHR = 1.82, 95% CI = 1.15–2.88; >65 y: aSHR = 1.39, 95% CI = 1.11–1.74) and those without comorbidities (aSHR = 1.41, 95% CI = 1.04–1.93). Comorbidities including diabetes, hypertension, heart failure, coronary artery disease (CAD), Stroke, and end stage renal disease (ESRD) significantly increased the risk of IBS.

**Conclusion:**

Patients hospitalized for SCI have increased risks of developing IBS. Though the mechanism that predisposes SCI patients to IBS is unclear, we suggest that physicians promptly identify and treat correctable risk factors.

## Introduction

Spinal cord injuries (SCI) are damages to the spinal cord due to traumatic causes such as road accidents, falls or violence and other causes including tumors and infections like extrapulmonary tuberculosis [1, 2]. According to the World Health Organization (WHO), the global incidence of SCI is estimated to be between 40 and 80 cases per million people. With increasing longevity, the prevalence has also increased in some countries. However, people with SCI still die at an earlier age than people without SCI [3–5].

Cardiovascular complications can occur after SCI with manifestations of bradyarrhythmia, orthostatic hypotension, vasodilation and venous stasis, impaired cardiovascular reflexes and autonomic dysreflexia [6]. SCI is associated with increased risk of atherosclerotic disease due to overweight condition, lipid disorders, metabolic syndrome and diabetes [7, 8]. Cardiovascular disease has been recognized to be the leading cause of morbidity and mortality in SCI patients [9]. In previous reports we demonstrated the association between SCI and myocardial infarction and acute coronary syndrome [10, 11].

Ischemic bowel syndrome (IBS) is a deadly life-threatening condition that can result in bowel infarction, sepsis and death and requires resection of the necrotic bowel, which can have further impact on the patient’s quality of life. IBS is caused by compromised blood flow to the involved segment of the bowel and can affect both the small and large intestines. Risks include cardiac emboli [12], need for hemodialysis [13, 14], inflammation, hypovolemia, and effects of chronic use of vasoconstrictive medications [15]. These risk factors were similar to complications observed in people with SCI. However, it is unclear whether SCI is associated with IBS.

To our knowledge, no studies have addressed the long-term risks of ischemic bowel syndrome in patients with SCI. In this study our aim was to determine whether SCI is associated with increased risk of IBS in an Asian cohort using data from the National Health Insurance Research Database (NHIRD), Taiwan. We also wanted to identify the co-morbidities and analyze the risks of developing ischemic bowel syndrome during follow-up.

## Methods

### Data Source

The National Health Insurance (NHI) program was launched in 1995 and includes all citizens living in Taiwan. The NHI program covers approximately 99% of citizens (about 23.75 million) and over 98% of the hospitals nationwide are under contract with the NHI [16]. The National Health Research Institutes (NHRI) established and maintained the NHIRD and released data annually for research purposes. The NHIRD contains medical information including drug prescriptions, and patient data including sex, date of birth, dates of visits or hospitalizations, and diagnoses coded using the International Classification of Diseases, Ninth Revision, Clinical Modification (ICD-9-CM) codes. For this retrospective cohort study, we used a subset of the NHIRD including files of inpatient claims and a registry of beneficiaries.

### Participant Sample

Patients aged ≥ 20 years in the inpatient database with newly identified spinal cord injury (SCI) (ICD-9 codes 806 and 952) from 2000 to 2011 were selected as the SCI cohort. The first-time SCI admission diagnosis date served as the index date. We divided SCI patients into subgroups: cervical-spine SCI (ICD-9-CM codes 806.0, 806.1, 952.0, 952.00, 952.01, 952.02, 952.03, 952.04, 952.05, 952.06, 952.07, 952.08, 952.09), thoracic-spine SCI (ICD-9-CM codes 806.2, 806.21, 806.26, 806.3, 952.1, 952.11, 952.16), lumbar, sacral, and coccygeal spine SCI (ICD-9-CM codes 806.4, 806.5, 806.6, 806.7, 806.8, 806.9, 952.2, 952.3, 952.4, 952.8, and 952.9) and multiple level SCI. We defined multiple level SCI as several SCI in one event (ICD-9-CM code 952.8).

Patients with a history of ischemic bowel syndrome (IBS) (ICD-9-CM 557.0, 557.1 and 557.9), less than 20 years of age and those with incomplete medical information were excluded. For each SCI patient, we randomly selected 4 persons without SCI from all NHI beneficiaries, and frequency-matched them according to age (every 5 year span), sex, the year of the index date, and comorbidities of diabetes, hypertension, hyperlipidemia, chronic obstructive pulmonary disease (COPD), coronary artery disease, heart failure, end stage renal disease (ESRD), stroke, and atrial fibrillation (AF) using the same exclusion criteria. A total of 42856 SCI patients and 171171 controls without SCI were enrolled in our study.

### Outcome and Co-morbidities

The main outcome was hospitalization with a new diagnosis of IBS during the follow-up. All subjects were followed up from the index date until the date of IBS diagnosis, withdrawal from the NHI program, or when the study period ended (December 31, 2011).

### Ethics Statement

The NHIRD encrypts patient personal information to protect privacy and provides researchers with anonymous identification numbers associated with relevant claims information, including sex, date of birth, medical services received, and prescriptions. Therefore, patient consent is not required to access the NHIRD. This study was approved to fulfill the condition for exemption by the Institutional Review Board (IRB) of China Medical University (CMUH104-REC2-115). The IRB also specifically waived the consent requirement.

### Data Availability Statement

All data and related metadata were deposited in an appropriate public repository in the National Health Research Institutes (NHRI). The data on the study population that were obtained from the NHIRD (http://nhird.nhri.org.tw/en/index.html) are maintained in the NHIRD (http://nhird.nhri.org.tw/). The NHRI is a nonprofit foundation established by the government. Only citizens of the Republic of China (Taiwan) who fulfill the requirements of conducting research projects are eligible to apply for the NHIRD. The use of NHIRD is limited to research purposes only. Applicants must follow the Computer-Processed Personal Data Protection Law (http://www.winklerpartners.com/?p=987) and related regulations of National Health Insurance Administration and NHRI, and an agreement must be signed by the applicant and his/her supervisor upon application submission. All applications are reviewed for approval of data release.

### Statistical Analysis

A Chi-square test and a Student’s t-test were used to evaluate the distribution of categorical and continuous variables, respectively, between the SCI and control cohorts. The Kaplan-Meier method was used to plot the cumulative incidences of IBS for the SCI cohort and the control cohort and a log-rank test was performed to examine the differences between the cohorts. The overall, sex-, age-, and co-morbidity-specific incidence densities of IBS were estimated for each cohort. By controlling the competing risk of death, the Fine and Gray model, which extends the standard univariable and multivariable Cox proportional-hazard regression model, was used to estimate the relative risk of IBS development in patients with SCI, compared with the non-SCI cohort. The multivariate models were simultaneously adjusted for age, sex, and co-morbidities, including diabetes, hypertension, hyperlipidemia, COPD, heart failure, CAD, stroke, ESRD and AF. A further analysis was performed to assess whether the association of IBS varied according to various levels of SCI. All analyses were performed using SAS version 9.3 (SAS Institute, Inc., Cary, NC, USA), with the significance level set at 0.05 in a 2-tailed test.

## Results

**Fig 1:**
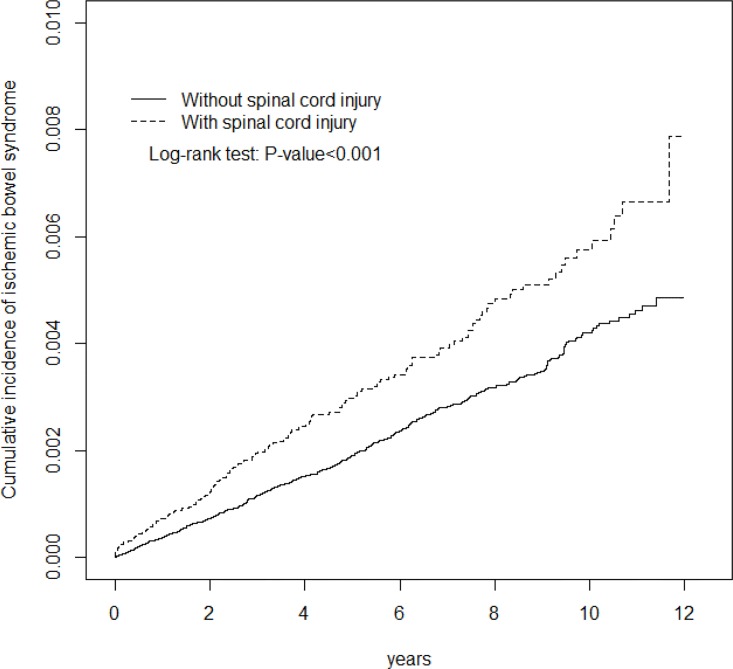
Cummulative incidence of ischemic bowel syndrome compared in patients with and without SCI using the Kaplan-Meier method.

The study retrospectively enrolled 42,856 SCI patients and 4-fold matched control subjects (n = 171,171) with a similar distribution of sex, age, and comorbidities (Table 1). In the SCI cohort, the majority of patients were male (63.3%) and those aged ≤49 years (46.5%). The mean ages of the SCI and control cohorts were 52.5 ± 18.3 and 52.2 ± 18.4 years, respectively. The major comorbidity in SCI cohort was hypertension (19.6%) in these study cohorts and followed by diabetes (12.5%), CAD (8.47%) and stroke (7.64%). The mean follow-up duration was 5.67 years for patients with SCI and 6.03 years for the control cohort. Fig 1 shows the cumulative incidence of IBS was significantly higher for patients with SCI than for control subjects (Log-rank test *P*< .001).

**Table 1:** Demographic characteristics and comorbidity in patient with and without Spinal cord injury.

	Spinal cord injury		
	No	Yes	
Variable	N = 171171	N = 42856	*p*-value
**Sex**	N (%)	N (%)	0.99
Female	62912(36.8)	15750(36.7)	
Male	108259(63.3)	27106(63.3)	
**Age, years**			0.99
≤49	79671(46.5)	19938(46.5)	
50–65	41926(24.5)	10494(24.5)	
>65	49574(29.0)	12424(29.0)	
mean(SD)	52.2(18.4)	52.5(18.3)	0.001^#^
**Comorbidity**			
Diabetes	21356(12.5)	5372(12.5)	0.74
Hypertension	33519(19.6)	8409(19.6)	0.85
Hyperlipidemia	7948(4.64)	2016(4.70)	0.59
COPD	10504(6.14)	2661(6.21)	0.58
Heart failure	5208(3.04)	1334(3.11)	0.45
CAD	14388(8.41)	3631(8.47)	0.66
Stroke	12975(7.58)	3273(7.64)	0.69
ESRD	870(0.51)	240(0.56)	0.18
AF	1528(0.89)	618(1.44)	0.25

Chi-Square Test

^#^: Two sample T-test.

In total, 145 SCI patients were identified as having IBS with an incidence rate of 5.97 per 10,000 person-years and 414 patients among the control cohort were diagnosed with IBS with an incidence rate of 4.01 per 10,000 person-years, yielding a crude SHR (cSHR) of 1.24 (95% CI = 1.03–1.49) and an adjusted SHR (aSHR) of 1.25 (95% CI = 1.04–1.51) (Table 2). The incidence of IBS in patients with SCI was 6.07 per 10,000 person-years in females and was up to 18.0 per 10,000 person-years in patients age >65 and those with co-morbidities the incidence reached as high as 14.5 per 10,000 person-years.

**Table 2:** Comparison of incidence and subhazard ratio of ischemic bowel syndrome stratified by sex, and age in patients with and without spinal cord injury.

	Spinal cord injury							
	No			Yes				
Variable	Event	PY	Rate^#^	Event	PY	Rate^#^	Crude SHR (95% CI)	Adjusted SHR^†^ (95% CI)
**All**	414	1032118	4.01	145	242861	5.97	1.24(1.03, 1.49)*	1.25(1.04, 1.51)*
**Sex**								
Female	228	383612	5.94	56	92332	6.07	0.88(0.66, 1.17)	0.90(0.68, 1.20)
Male	186	648506	2.87	89	150529	5.91	1.45(1.14, 1.83)**	1.47(1.16, 1.86)**
**Stratified by age**								
≤49	29	524529	0.55	19	128716	1.48	2.18(1.26, 3.78)**	2.15(1.24, 3.74)**
50–65	58	250488	2.32	26	58465	4.45	1.80(1.14, 2.85)*	1.82(1.15, 2.88)*
>65	327	257370	12.7	100	55681	18.0	1.39(1.11, 1.73)**	1.39(1.11, 1.74)**
**Comorbidity‡**								
No	116	747742	1.55	52	178660	2.91	1.25(0.92, 1.69)	1.41(1.04, 1.93)*
Yes	298	284376	10.5	93	64201	14.5	1.17(0.94, 1.47)	1.23(0.98, 1.54)

Rate^#^, incidence rate, per 10,000 person-years; Crude SHR, crude subhazard ratio.

Adjusted SHR†: multivariable analysis including age, sex, and co-morbidities (diabetes, hypertension, hyperlipidemia, COPD, heart failure, CAD, stroke, ESRD, and AF).

*p<0.05

**p<0.01.

Comorbidity‡: Patients with any one of the following co-morbidities, diabetes, hypertension, hyperlipidemia, COPD, heart failure, CAD, stroke, ESRD, and AF were classified as the co-morbidity group.

Multivariate analysis showed that individuals with SCI were associated with a greater risk of IBS than individuals without SCI among males, all age groups, and those without co-morbidities (aSHR = 1.41, 95% CI = 1.04–1.93). In the multivariate model, the risk of IBS was increased increased with increasing age (every year) (aSHR = 1.01, 95% CI = 1.01–1.02) (Table 3). We also observed that diabetes, hypertension, heart failure, CAD, and ESRD significantly influenced the risk of IBS. Compared with patients without SCI, patients with T-spine injury (aSHR = 1.50, 95% CI = 1.02–2.21) (Table 4).

**Table 3:** Subhazard ratios of ischemic bowel syndrome in association with gender, age and co-morbidities in competing risk (death) models.

	Crude		Adjusted^†^	
Variable	SHR	(95% CI)	SHR	(95% CI)
**Sex (male vs female)**	1.01	(0.87, 1.18)	0.96	(0.82, 1.12)
**Age, years**	1.02	(1.01, 1.02)***	1.01	(1.01, 1.02)***
**Baseline comorbidities (no vs yes)**				
Diabetes	1.93	(1.60, 2.33)***	1.37	(1.12, 1.67)***
Hypertension	2.65	(2.24, 3.15)***	1.78	(1.46, 2.17)***
Hyperlipidemia	1.78	(1.36, 2.34)***	0.97	(0.73, 1.30)
COPD	2.13	(1.68, 2.70)***	1.29	(1.00, 1.66)
Heart failure	3.77	(2.90, 4.88)***	1.90	(1.41, 2.56)***
CAD	2.64	(2.16, 3.21)***	1.45	(1.15, 1.83)**
Stroke	2.36	(1.91, 2.92)***	1.46	(1.16, 1.83)**
ESRD	8.80	(5.64, 13.7)***	5.27	(3.34, 8.31)***
AF	2.78	(1.84, 4.19)***	1.38	(0.90, 2.12)

Crude SHR, relative subhazard ratio; Adjusted SHR^†^: multivariable analysis including age, and co-morbidities (diabetes, hypertension, hyperlipidemia, COPD, heart failure, CAD, stroke, ESRD and AF).

**p<0.01

***p<0.001.

**Table 4:** Incidence, and hazard ratio of ischemic bowel syndrome among patients with different level spine injuries.

Variable	Event	PY	Rate^#^	Crude SHR (95% CI)	Adjusted SHR^†^ (95% CI)
**Non-SCI**	414	1032172	4.01	1(Reference)	1(Reference)
**C-spine injury**	55	125374	4.39	0.85(0.65, 1.12)	0.96(0.73, 1.26)
**T-spine injury**	27	27895	9.68	1.58(1.08, 2.32)*	1.50(1.02, 2.21)*
**L-S-Co-spine injury**	48	71098	6.75	1.20(0.90, 1.61)	1.21(0.90,1 .62)
**Multiple spine level injury**	15	18441	8.13	1.47(0.88, 2.45)	1.54(0.92, 2.57)

Rate^#^, incidence rate, per 10,000 person-years; Crude SHR, crude subhazard ratio; Adjusted SHR^†^: multivariable analysis including age, sex, and co-morbidities (diabetes, hypertension, hyperlipidemia, COPD, heart failure, CAD, stroke, ESRD and AF).

*p<0.05.

C-spine SCI: ICD-9-CM 06.0, 806.1, 952.0, 952.00, 952.01, 952.02, 952.03, 952.04, 952.05, 952.06, 952.07, 952.08, 952.09.

T-spine SCI: ICD-9-CM 806.2, 806.21, 806.26, 806.3, 952.1, 952.11, 952.16.

L-S-Co-spine SCI: ICD-9-CM 806.4, 806.5, 806.6, 806.7, 806.8, 806.9, 952.2, 952.3, 952.4, 952.8, and 952.9.

C: cervical, T: thoracic, L-S-Co: lumbar, sacral, and coccygeal; SCI: spinal cord injury.

## Discussion

To the best of our knowledge, this is the first study to show an association between SCI and ischemic bowel syndrome. We identified 145 IBS patients from a total of 42856 patients hospitalized for SCI from 2000 to 2011 by analyzing claims data from the NHIRD in Taiwan. Patients with SCI have higher risks of developing IBS, with an aSHR of 1.25, 95% CI = 1.04–1.51). Co-morbidities including diabetes, hypertension, heart failure, CAD, and ESRD significantly influenced the risk of IBS.

IBS can be divided into mesenteric ischemia (MI) and ischemic colitis (IC). While IC is mostly self-limited owing to the communications between inferior mesenteric arteries and the internal iliac arteries, MI is more devastating and fatal as no communicating arteries can help during ischemia. Although IC is usually preceded by an episode of transient hypoperfusion and MI follows SMA occlusion either by embolic event or atherosclerotic change, MI and IC can share similar risks. ESRD was thought to be one of the risks for IS, but in an analysis from the Taiwan NHIRD for 1998–2007, the risk of MI for patients with ESRD was higher than the general population [17]. Another population-based case control study conducted in the UK showed that heart failure were associated with IC rather than MI, while diabetes and prior cardiovascular surgery were associated with higher risk of MI, but not with IC [18].

In the present study we identified patients with IBS according to the ICD-9 CM codes used in the NHIRD claims data. The definition of ICD-9-CM could not properly differentiate between patients with MI or IC. It was not possible to review all the medical records to verify if a patient had truly MI or IC. In the clinical practice the patients with severe abdominal pain could have both MI and IC and it is difficult to properly separate MI from severe IC. And patients with IC might not present with prominent features or hospitalized and thus could be underestimated in the cohort.

SCI patients, including our cohort, were shown to have increased risks of cardiovascular events and more prevalent cardiovascular co-morbidities and other co-morbidities including ESRD. In the present study, diabetes, hypertension, heart failure, CAD, and ESRD influenced the risk of developing IBS. The exact mechanism of SCI that leads to IBS is still unclear but it is possible that these comorbid conditions may ultimately lead to development of IBS.

Patients with SCI may not present with typical pain characteristics below the lesion level. One of the major cardiovascular complications is autonomic dysreflexia, which occurs in patients with SCI at level T6 or above. The breakdown of communication between the brainstem and autonomic nervous system may lead to sympathetic hyperactivity with vasoconstriction below the neurological lesion level when a noxious stimulus exists such as bladder distention or fecal impaction. Besides, orthostatic hypotension, severe arterial hypotension, bradycardia, impaired function of baroreceptors, hyponatremia and low plasma volume also occur and motor deficit and recumbence leading to loss of skeletal muscle pumping activity will ultimately lead to vascular dysfunction [19]. Low blood flow rate will lead to chronic hypoperfusion of the intestine which further contribute to the development of IBS. We hypothesize that vasoconstriction below the level of injury and complicated systemic hypotension and low blood flow rate may lead to decreased perfusion of the intestine and ultimately lead to IBS irrespective of the cardiovascular co-morbidities that also increase the risk of IBS.

The strength of this study is that the sample size is large enough to enable meaningful analysis and subgroup analysis. There were 42856 patients newly identified with SCI from 2000 to 2011 among nearly 23.75 million citizens with an estimated incidence of 150 cases per million people, which is higher than the global incidence. We focused on the relationship between SCI and IBS and found the association between SCI and IBS was unclear and autonomic dysreflexia could be associated with the development of IBS. Nevertheless, it is important to identify and adjust correctable cardiovascular risks which increase the risks of IBS. Therefore we recommend correctable risk control in patients with SCI.

There were several inherent limitations in the administrative data. First, we chose a retrospective design and used ICD-9 CM codes without angiographic confirmation. Second, we couldn’t conduct a review of medical records and additional patient information about smoking, alcohol consumption, body mass index, family history of IBS, and the severity of IBS was not available in this dataset. Third, we didn’t try to identify medications which could also influence the development of IBS. Furthermore, we excluded patients who were not hospitalized and we might have excluded those patients with non-occlusive ischemia or transient form of ischemic colitis who might have less severe symptoms.

In conclusion, we have demonstrated that patients hospitalized for SCI have increased risks of IBS. Though the mechanism that predisposes SCI patients to IBS is unclear, it is possible that IBS in these patients may have obscure symptoms. We suggest that physicians promptly identify and treat correctable risk factors.

## Supporting Information

S1 STROBE Checklist Checklist of items that should be included in reports of observational studies. (DOC)

S1 Table ICD-9-CM codes in the study. (PDF)

S2 Table Incidence, and hazard ratio of ischemic bowel syndrome among patients with different level and completeness of spine injuries. (PDF)

## Abbreviations

aSHR: adjusted subhazard ratio
CI: confidence interval
NHIRD: National Health Insurance Research Database
NHI: National Health Insurance
NHRI: National Health Research Institutes
LHID 2000: Longitudinal Health Insurance Database 2000
SCI: Spinal cord injuries
IBS: Ischemic bowel syndrome
COPD: Chronic obstructive pulmonary disease
CAD: Coronary artery disease
ESRD: End stage renal disease.
