# Supplementary material for: Ischemic Bowel Syndrome in Patients with Spinal Cord Injury: A Nationwide Study
Source: PLoS One. 2017 Jan 5;12(1):e0169070. doi: 10.1371/journal.pone.0169070 (PMC5215787; doi:10.1371/journal.pone.0169070)
Supplement: S1 Table — (PDF) [file pone.0169070.s002.pdf]

S1 Table. ICD-9-CM codes in the study.

| **ICD-9-CM codes** | **Diagnosis** |
|---|---|
| 806, 952 | Spinal cord injury |
| 557.0, 557.1 and 557.9 | Ischemic bowel syndrome |
| 250 | Diabetes |
| 401-405 | Hypertension |
| 272 | Hyperlipidemia |
| 491, 492, 496 | COPD |
| 428 | Heart failure |
| 410-414 | CAD |
| 430-438 | Stroke |
| 585 | ESRD |
| 427.31 | AF |
