# Supplementary material for: Ischemic Bowel Syndrome in Patients with Spinal Cord Injury: A Nationwide Study
Source: PLoS One. 2017 Jan 5;12(1):e0169070. doi: 10.1371/journal.pone.0169070 (PMC5215787; doi:10.1371/journal.pone.0169070)
Supplement: S2 Table — (PDF) [file pone.0169070.s003.pdf]

S2 Table. Incidence, and hazard ratio of ischemic bowel syndrome among patients with different level and completeness of spine injuries.

Variable	Event	PY	Rate#	Crude SHR (95% CI)	Adjusted SHR† (95% CI)
**Non-SCI**	414	1032118	4.01	1(Reference)	1(Reference)
C-Spine complete SCI	2	3870	5.17	0.98(0.24, 3.91)	1.07(0.27, 4.30)
C-Spine incomplete SCI	26	61628	4.22	0.79(0.54, 1.17)	0.90(0.61, 1.33)
T-Spine complete SCI	0	1105	0.00	-	-
T-Spine incomplete SCI	3	2142	14.0	2.46(0.79, 7.65)	2.52(0.81, 7.85)
L-S-C-Spine complete SCI	0	179	0.00	-	-
L-S-C-Spine incomplete SCI	10	17827	5.61	1.10(0.59,2 .05)	1.12(0.60, 2.10)
unspecified SCI	89	137669	6.46	1.18(0.94, 1.47)	1.21(0.96, 1.51)
Multiple SCI at one visit	15	18441	8.13	1.47(0.88, 2.45)	1.53(0.92, 2.56)

Rate#, incidence rate, per 10,000 person-years; Crude SHR, crude subhazard ratio; Adjusted SHR†: multivariable analysis including age, sex, and co-morbidities (diabetes, hypertension, hyperlipidemia, COPD, heart failure, CAD, stroke, ESRD and AF)

C-Spine complete SCI: 806.01, 806.06, 806.11, 806.16, 952.01, 952.06

C-Spine incomplete SCI: 806.00, 806.02, 806.03, 806.04, 806.05, 806.07, 806.08, 806.09, 806.10, 806.12, 806.13, 806.14, 806.15, 806.17, 806.18, 806.19, 952.00, 952.02, 952.03, 952.04, 952.05, 952.07, 952.08, 952.09

T-Spine complete SCI: 806.21, 806.26, 806.31, 806.36, 952.11, 952.16

T-Spine incomplete SCI: 806.20, 806.22, 806.23, 806.24, 806.25, 806.27, 806.28, 806.29, 806.30, 806.32, 806.33, 806.34, 806.35, 806.37, 806.38, 806.39, 952.10, 952.12, 952.13, 952.14, 952.15, 952.17, 952.18, 952.19

L-S-C-Spine complete SCI: 806.61, 806.71

L-S-C-Spine incomplete SCI: 806.4, 806.5, 806.60, 806.62, 806.69, 806.70, 806.72, 806.79, 952.2, 952.3, 952.4

Unspecified SCI: 806, 806.0, 806.1, 806.2, 806.3, 806.6, 806.7, 806.8, 806.9, 952, 950.0, 952.1, 952.9
